# From Insight to Impact: Advancing Learning Health System Capability Through a System-Wide Quality Improvement Symposium

**DOI:** 10.7759/cureus.102976

**Published:** 2026-02-04

**Authors:** Anna Dermenchyan, Danielle S Seiden, Manisha Bhayani, Angela Song, Namrata Venkatesan, Khushi Sharma, Kavya Pandrangi, Maria A Han

**Affiliations:** 1 Medicine, University of California Los Angeles, Los Angeles, USA; 2 Quality and Patient Safety, University of California Los Angeles, Los Angeles, USA; 3 Ambulatory Care, University of California Los Angeles, Los Angeles, USA

**Keywords:** behavioral science, continuing education, healthcare quality improvement, interprofessional education, learning health system, narrative-based education, organizational culture, program evaluation, quality improvement education, systems learning

## Abstract

As healthcare systems navigate increasing complexity, the need to embed quality improvement (QI) into organizational culture has never been greater. In alignment with national trends emphasizing a shift from compliance-driven quality to shared responsibility and excellence, the University of California Los Angeles (UCLA) Health launched its inaugural Quality Symposium, led by the Department of Medicine. The event aimed to cultivate a Learning Health System community by strengthening shared learning, accelerating system improvement, and increasing the visibility and adoption of QI across clinical settings. The symposium featured a keynote presentation on artificial intelligence (AI) and healthcare transformation, followed by nine Technology, Entertainment, Design (TED)-style talks organized across three modules: ambulatory care quality, inpatient care quality, and research and education. Attendees completed pre- and post-event evaluations assessing satisfaction, perceived learning, and actionable takeaways. The symposium drew 218 registrants and approximately 160 attendees (73%), both in person and online, including physicians, nurses, administrators, and trainees. Among post-survey respondents (62 of approximately 160 attendees, 39%), 52 (88%) reported gaining at least one actionable insight applicable to their work. Qualitative analysis identified themes emphasizing the value of interprofessional storytelling, visible leadership engagement in QI initiatives, and the integration of behavioral science and technology to support improvement efforts. This learning report describes the design, implementation, and perceived educational impact of a health system-wide symposium, framed as a descriptive program evaluation. Findings suggest that narrative-driven education, behavioral insight, and cross-disciplinary engagement may support system learning and collaboration within academic health systems.

## Introduction

Healthcare leaders today are navigating competing demands, including operational efficiency pressures, workforce shortages, rising costs, and the rapid integration of emerging technologies [1]. The National Association for Healthcare Quality (NAHQ) 2025 State of Healthcare Quality report describes this landscape as one of opportunity: "while challenges persist, the perception of quality is evolving from a compliance function to a shared, strategic imperative that drives safety, equity, efficiency, and organizational excellence" [2]. This reframing aligns with Learning Health System frameworks and the Quadruple Aim, which view quality as a central organizational function that informs care design, technology adoption, and clinician support, rather than a set of regulatory requirements [3-5].

Healthcare Quality Week (HQW), established by the NAHQ, is an annual recognition event that celebrates the contributions of healthcare quality and safety professionals across the continuum of care [6]. Observed each October, HQW highlights the critical role of quality improvement (QI) in advancing patient safety, clinical excellence, and organizational performance. The week serves as an opportunity for institutions to reflect on progress, share best practices, and honor the interprofessional teams that drive continuous improvement in healthcare delivery. Although launched as a recognition week, HQW also operates as a mechanism for health systems to spread effective practices, promote interprofessional learning, and reinforce shared improvement priorities [7]. In this context, HQW functions not only as a celebration but as a strategic tool for alignment, strengthening engagement, supporting a learning-oriented culture, and advancing NAHQ's vision of quality as a core institutional priority.

At the University of California Los Angeles (UCLA) Health, the Department of Medicine (DoM) Quality Program has long emphasized quality as a core driver of institutional performance and learning. The program integrates data analytics, behavioral science, and clinician engagement to strengthen care delivery across ambulatory and inpatient settings. In parallel with these operational efforts, DoM Quality has increasingly focused on building a shared learning infrastructure and cultivating system-wide improvement capability. Recognizing the importance of expanding this work through structured knowledge exchange and interprofessional engagement, the DoM hosted its inaugural Quality Symposium during Healthcare Quality Week 2025 [8].

Although led by the DoM Quality Program, the symposium was intentionally designed to engage health system leaders, clinicians, staff, trainees, and external partners, reflecting a health system-wide approach to learning and improvement. The event's theme, "The Pulse of Progress: Why Quality Is Healthcare's Vital Sign", reflected a mission to make quality and safety visible, accessible, and actionable. Beyond showcasing programmatic achievements, the symposium emphasized reflection, storytelling, and community building around improvement. By translating ongoing quality work into shared understanding, practical application, and cultural reinforcement, the symposium sought to strengthen learning health system capability across the Department of Medicine and UCLA Health.

Despite widespread adoption of QI education and system-wide learning initiatives, there is limited published literature describing how large-scale, interprofessional symposia are designed, implemented, and evaluated as mechanisms for advancing Learning Health System capability within academic medical centers. This learning report addresses this gap by offering a descriptive evaluation of a system-wide quality improvement symposium, with attention to feasibility, participant engagement, and perceived educational value rather than causal or longitudinal outcomes. The aim of this manuscript is to describe a health system-wide quality improvement symposium as a program evaluation (learning report), summarize its structure, implementation, and lessons learned, and highlight a scalable model for strengthening quality improvement capability and culture in academic medical centers.

## Materials and methods

Design and objectives

The symposium was designed as a half-day, in-person event at UCLA Health's Ronald Reagan Medical Center on October 22, 2025. The goals were to: (1) highlight innovative QI strategies across care settings; (2) promote interprofessional collaboration and shared accountability; and (3) foster reflection, learning, and inspiration among clinicians, leaders, and staff to advance a learning health system. The symposium was also intended to align health system QI efforts with the national conversation during HQW, reinforcing the health system's commitment to continuous improvement.

This evaluation was intentionally designed as a pragmatic, descriptive learning report, rather than a hypothesis-driven effectiveness study. Accordingly, outcome measures focused on participant characteristics, engagement, and self-reported learning and perceived applicability, rather than objective behavioral change or system-level outcomes. The methods are intended to support transparency, contextual understanding, and hypothesis generation for similar educational interventions within other academic health systems.

Structure and format

The program opened with welcome remarks from departmental and health system leaders, including the Interim Chief Quality Officer of the Department of Medicine, the Chair and Executive Medical Director of the Department of Medicine, the Chief Medical Officer for Ambulatory Care, and the Chief Medical and Quality Officer for UCLA Health. These remarks emphasized that quality is not a stand-alone initiative but a unifying principle for academic health systems and organizational transformation.

The keynote presentation explored the potential of artificial intelligence (AI) to augment clinicians' ability to deliver high-quality, efficient, and compassionate care. The symposium then transitioned into nine short, Technology, Entertainment, Design (TED)-style talks delivered by DoM Quality team members and collaborators across three thematic modules: (1) ambulatory care quality which aligns performance incentives, improving cardiovascular care, and expanding team-based models to reduce clinician burnout; (2) inpatient care quality which innovates in physician engagement, mortality review, and hospital avoidance through the Next Day Clinic; and (3) research and education which are student-led QI innovation, peer coaching, and behavioral science applications in healthcare. Each thematic module concluded with a facilitated panel discussion integrating questions from in-person and virtual attendees.

**Figure 1:**
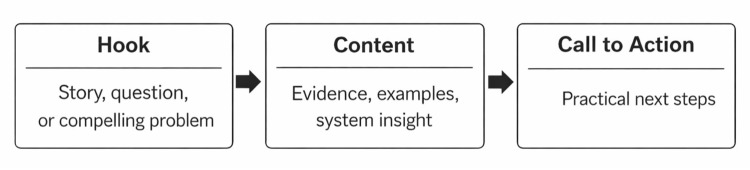
Structured presentation framework used in the Department of Medicine Quality Symposium This figure illustrates the structured presentation framework used to guide symposium speakers. Presentations were designed to begin with a Hook to engage attention, followed by focused Content grounded in evidence and real-world examples, and to conclude with a Call to Action highlighting practical steps participants could apply in their own settings.

To ensure coherence and engagement, presenters were guided to follow a structured, commonly used TED-inspired presentation approach built around Hook - Content - Call to Action, prioritizing clarity and storytelling over data-heavy reporting (Figure 1). Each talk began with a Hook, such as a compelling story, a surprising statistic, or a relatable clinical challenge, to highlight the importance of the topic. The Content section provided a focused explanation of the central idea, supported by evidence and real-world examples arranged in a logical sequence. Presentations concluded with a Call to Action offering clear, actionable steps attendees could apply within their own settings. This design ensured that presentations were concise, memorable, and oriented toward practical impact [9]. To further support the shift from traditional academic presentations toward formats that are easily shareable and engaging, PechaKucha was shared as an example of a tightly structured, visually driven storytelling approach that can enhance attention and efficiency in conference settings [10]. PechaKucha is a presentation format characterized by a fixed number of visually focused slides delivered in rapid succession, designed to promote clarity, brevity, and audience engagement.

To further enhance the symposium's educational value, a Continuing Nursing Education (CNE) process was implemented in collaboration with the UCLA Department of Nursing, offering 4.5 contact hours to nurses who attend the full program. Providing formal continuing education credit was an intentional strategy to increase engagement and support QI-centered professional development among participants. The planning team also explored offering Continuing Medical Education (CME) credit; however, the cost and administrative requirements exceeded available resources, making CNE the most feasible and impactful option for this inaugural event.

Participants and evaluation

A total of 218 individuals registered for the symposium, including physicians, nurses, staff, and trainees from the Department of Medicine, as well as participants from UCLA Health, other UCLA schools and departments, and external organizations. Because the event was open to the public, additional attendees included representatives from partner health systems and community stakeholders.

Evaluation methods included pre- and post-symposium surveys. The pre-symposium survey, embedded in the event registration, assessed baseline familiarity with QI methods, prior QI involvement, motivations for attending, and institutional role or affiliation. After the event, participants were invited to complete a general evaluation survey. The pre- and post-event survey instruments were developed by the authors specifically to evaluate the effectiveness of the symposium. Content validity was established through internal review and iterative refinement. The full questionnaires are available in Appendices A and B, respectively. For those seeking CNE credit, the nursing-specific evaluation was a standard UCLA Department of Nursing CNE evaluation instrument (not previously published) and was used as part of the continuing education process. Both tools measured perceived learning, the applicability of QI concepts, and overall satisfaction, although the items differed slightly due to accreditation requirements. A combination of Likert-scale items and open-ended questions enabled the collection of both quantitative responses and narrative insights [11]. The primary outcome was defined as high QI knowledge or ability, operationalized as reporting "very familiar" or "somewhat familiar" with QI methods on the pre-survey and "strongly agree" or "agree" with increased QI understanding or the ability to apply QI concepts on the post-surveys. Since post-surveys were optional and anonymous, they included both general attendees and nurses. 

Ethical considerations

This project was conducted as a quality improvement and educational program evaluation using de-identified, aggregate survey data. In accordance with UCLA Health policy, the activity did not meet criteria for human subjects research and was therefore exempt from Institutional Review Board review.

## Results

As expected for a voluntary educational symposium, attendees represented a highly engaged audience with prior interest and experience in quality improvement, which should be considered when interpreting the findings.

Pre-event survey findings

A total of 218 individuals registered and completed at least one pre-event survey item; item-level response counts ranged from 207 to 218. Respondents included both general attendees and nurses seeking CNE credit. Most respondents were UCLA-affiliated (176, 82%), with roles including staff (120, 68%), faculty (43, 24%), trainees (8, 5%), and others (5, 3%). Email was the primary communication channel through which participants learned about the symposium (117, 54%), followed by organizational announcements (48, 22%) and peer referrals (37, 17%).

As shown in Table 1, respondents were a highly engaged audience with substantial QI experience. Nearly three-quarters reported prior involvement in QI initiatives (152, 73%), with 98 (47%) prospective attendees having led a QI project and 54 (26%) having contributed to one. An additional 42 respondents (20%) expressed interest in participating in future QI efforts. Familiarity with QI methods was similarly high: Ninety-three participants (45%) regularly used QI tools, 78 (38%) had some exposure, and 36 (17%) were new to QI. 

**Table 1:** Distribution of pre-symposium responses on QI experience, QI familiarity, and attendance motivations * QI Experience and QI Familiarity items were completed by 207 respondents; ** Motivation items are based on all 218 pre-survey respondents, with multiple responses allowed QI - quality improvement; UCLA - University of California Los Angeles

Category	Metric	N (%)
QI Experience*	Yes, I have led a QI project	98 (47%)
	Yes, I have participated in a QI project	54 (26%)
	Not yet, but I am interested	42 (20%)
	No, and I'm not sure	13 (6%)
QI Familiarity*	Very familiar - I use them regularly	93 (45%)
	Somewhat familiar - I've been exposed but don't use them often	78 (38%)
	Not familiar - this is new to me	36 (17%)
Motivation**	Learn new tools/strategies	168 (77%)
	Hear about successful projects	143 (66%)
	Learn about current UCLA Health QI initiatives	140 (64%)
	Network with colleagues	134 (62%)
	Explore opportunities to get involved in QI	105 (48%)
	Other	5 (2%)

Participants' motivations for attending centered on professional growth and collaboration. The most frequently selected reasons included learning new tools and strategies (168, 77%), hearing about successful projects (143, 66%), learning about current UCLA Health QI initiatives (140, 64%), networking with colleagues (134, 62%), and exploring opportunities to engage in QI (105, 48%). More than one-third of registrants selected all five motivations, underscoring strong enthusiasm for applied learning and community-building around quality and patient safety.

Open-ended responses echoed this enthusiasm, highlighting participants' interest in continuing education opportunities, practical applications of QI (including AI and performance metrics), and broader institutional engagement. Respondents also expressed appreciation for the event and a desire for ongoing initiatives to connect and contribute to UCLA Health's quality improvement ecosystem.

Symposium content and thematic focus

To complement the quantitative results and to contextualize the learning outcomes, thematic summaries of each session are included in Table 2, illustrating the symposium's conceptual breadth and recurring emphases. Across the keynote and three thematic blocks, presenters highlighted shared insights related to data-driven improvement, interprofessional collaboration, system redesign, and behavioral approaches to advancing quality. Together, these themes represent the core content to which participants were exposed and provide essential context for interpreting subsequent quantitative and qualitative findings related to learning and engagement.

**Table 2:** Core Themes and Conceptual Emphases Across Symposium Presentations

Theme	Learnings
Keynote (Artificial Intelligence & Quality)	Highlighted how AI can serve as a catalyst for advancing healthcare quality, safety, and equity [12-13]. Emphasized that AI should augment, not replace, clinician judgment, acting as a “co-pilot” to enhance accuracy, efficiency, and patient experience [14-15]. Highlighted the importance of responsible data governance, workflow integration, and ethical oversight to prevent bias and promote trust [16]. Underscored that technology should be purpose-driven, strengthening systems of care rather than serving as innovation for its own sake [12].
Ambulatory Care Quality	Demonstrated how transforming quality metrics into clinical insights links measurement to meaningful outcomes [17-18]. Showed that combining data transparency, frontline engagement, and incentive-based programs strengthens adherence to evidence-based care [19]. Highlighted the value of peer collaboration and technology-enabled outreach in closing cardiovascular and chronic disease care gaps [20]. Showed that team-based care models can reduce clinician burnout, improve access, and enhance continuity, supporting both patient outcomes and provider well-being [21-23].
Inpatient Care Quality	Illustrated how building a shared inpatient quality portfolio aligns priorities, clarifies goals, and drives improvement [24]. Demonstrated the impact of rapid mortality review as a real-time, non-punitive learning process that strengthens safety culture [25]. Showed how reflective debriefings convert adverse outcomes into system-wide learning and team resilience [25]. Highlighted the Next Day Clinic as an innovative hospital avoidance initiative that reduces ED strain and hospital bed utilization [26].
Research & Education	Highlighted the undergraduate THINQ program as a model for cultivating early QI skills, systems thinking, and experiential learning among emerging professionals [27-28]. Demonstrated how peer coaching builds clinician well-being, fosters belonging, and strengthens professional growth [29]. Showed how behavioral science interventions (“nudges”) can make evidence-based practices easier to adopt for both clinicians and patients [30]. Collectively emphasized that advancing quality depends not only on strong systems and data but also on developing people to lead improvement in a learning health system [31].

Learning outcomes

The symposium achieved strong engagement and meaningful learning outcomes among attendees (Table 3). Of the 62 participants (General attendees N=35 (56%); Nurse attendees N=27 (44%)) who completed post-survey evaluations, 52 (88%) reported gaining at least one actionable idea they could apply in their own work, and 58 (94%) rated the overall quality of the event as excellent. 

**Table 3:** Summary of participant responses on actionable learning and overall symposium quality

Category	Question	Audience	N (%)
Total responses		General	35 (56%)
		Nurses	27 (44%)
		Total	62
Actionable insights	"I gained at least one actionable insight or idea that I can apply in my own work." Highest rating	General	28/32 (88%)
	"How would you rate your ability to apply what you learned?" Highest rating	Nurses	24/27 (89%)
	Combined score	Total	52/59 (88%)
Overall event rating	"How would you rate your overall experience at the DoM Quality Symposium?" Highest rating	General	33/35 (94%)
	"How would you rate the quality of this Event/Presentation/Training?" Highest rating	Nurses	25/27 (93%)
	Combined score	Total	58/62 (94%)

Post-event qualitative feedback demonstrated the symposium's effects on strengthening Learning Health System capabilities (Table 4). Attendees described a newly acquired understanding of data-driven approaches, structured improvement methods, and real-time feedback mechanisms, as well as intentions to adapt presented initiatives within their own clinical contexts. Responses also highlighted growing attention to behavioral science strategies and emerging technologies, including AI, as tools to support behavior change and patient communication. Importantly, participants identified both opportunities for cross-departmental collaboration and structural barriers, such as limited protected time for QI work, emphasizing the need for organizational support to sustain Learning Health System capabilities.

**Table 4:** Key themes from qualitative feedback and their implications for quality improvement and Learning Health System development QI - quality improvement; AI - artificial intelligence

Theme	Representative quotes	Implication for QI practice
Importance of data-driven QI	"From the keynote speaker to all the presenters, one insightful idea stood out to me: utilizing data is the most crucial element in any quality improvement or systemic enhancement effort." "Collect data so you know what to focus on."	Demonstrates understanding that clear measurement and routine use of data are central to QI.
Workforce capability building	"Incorporating a structured approach to my improvement projects." "I really enjoyed learning about the Rapid Mortality Review process and am hoping to bring some of that knowledge to our pediatric mortality review process."	Demonstrates that symposiums can build durable QI capability beyond awareness.
Culture of reflection and learning	"... I learned the value of real time feedback and debrief." "There is always room to improve."	Indicates movement toward a learning-oriented organizational culture.
Behavioral science as an enabler	"Consider phrasing for how to motivate behavior change." "Behavioral psychology tips."	Suggests readiness to apply evidence-based behavior change frameworks in QI initiatives.
Technology and AI as augmenting tools	"I enjoyed learning how to use AI to assist with care but not taking over." "AI-generated patient education and communication."	Aligns technology adoption with responsible innovation in a Learning Health System.
Structural barriers to QI	"No support from the management to carve out a time to QI project by the team."	Highlights need for institutional investment to sustain improvement capability.

Operational lessons

The planning process itself generated meaningful operational learning for the organizing team. Coordinating across multiple departments, including Health System Quality, Department of Nursing, and Information Technology, highlighted the importance of shared infrastructure and collaborative planning in executing a large-scale educational event. Providing storytelling coaching for presenters proved valuable, helping transform technical quality reports into clear, compelling, and human-centered narratives. Offering CNE credit enhanced nursing engagement and encouraged full-session participation. Offering CME credit was explored but not feasible for this inaugural event due to cost and administrative requirements. We therefore prioritized relevance and applicability by designing the symposium around clinicians' professional values and practical, actionable quality-improvement takeaways.

Several additional challenges emerged during planning and implementation. First, the symposium was initially advertised as an in-person event, but a webinar link was added the day prior to accommodate registrants with limited availability. This last-minute adjustment created some logistical complexity, and remote participants expressed interest in more robust virtual access in the future. Second, significant staff and student volunteer support was required to coordinate the event amid competing operational and clinical priorities, underscoring the need for additional infrastructure to sustain the symposium annually. Third, the narrative-based presentation format, central to the TED-style design, challenged presenters to depart from traditional academic styles and develop story-driven slide decks, requiring additional coaching and preparation time. These operational lessons are now being incorporated into planning for next year's symposium to support smoother workflows and an even more engaging participant experience.

## Discussion

Symposium attendees reported increased confidence and actionable takeaways. Participants described feeling more confident applying QI tools and concepts after the event, suggesting that the symposium's narrative-driven, cross-disciplinary format supported readiness to engage in improvement work. Although the pre- and post-survey samples were not identical and outcomes were self-reported, the overall pattern indicates that a structured, interprofessional learning environment can influence perceived knowledge and confidence over a short, concentrated time frame. These findings highlight the potential role of health system-level educational interventions in fostering QI capability, even within large and complex academic health systems. Importantly, the results reflect perceived learning and readiness rather than demonstrated changes in behavior, clinical outcomes, or system performance, and should be interpreted as exploratory and hypothesis-generating.

The NAHQ 2025 report emphasizes that healthcare quality is now recognized as a shared responsibility, not the domain of a select few. The DoM Quality Symposium directly exemplified this shift. By bringing together stakeholders across roles-clinicians, staff, students, administrators, and leaders-the event operationalized what NAHQ describes as "moving from compliance to excellence" [2,6,8]. The leadership engagement further reinforced the idea that quality must be embedded in systems, processes, and culture rather than existing as an isolated metric. This aligns closely with NAHQ's finding that 94% of healthcare leaders now link quality directly with patient safety and excellence [2]. 

The convergence between national trends and the themes emphasized in this local symposium underscores how institutional initiatives can operate as pivotal mechanisms for translating system-level priorities into frontline practice. Prior work has shown that structured QI programs are uniquely positioned to catalyze cultural change, particularly when they make abstract national goals tangible for clinicians through concrete tools, shared language, and actionable frameworks [32]. Similarly, the Learning Health System model highlights how local data infrastructures and collaborative learning environments support continuous adaptation and accelerate the uptake of evidence-based practices across care settings [33]. Our experience suggests that health system-level symposia function as practical vehicles for these processes by convening interdisciplinary stakeholders, reinforcing shared standards, and showcasing replicable improvement strategies. More broadly, this approach offers a roadmap for other health systems: when local initiatives are intentionally aligned with national priorities, they can strengthen organizational learning, reduce variation in practice, and advance quality across the continuum of care.

A defining element of the symposium was the TED-style storytelling format. Rather than focusing solely on metrics, presenters contextualized their projects through human stories; for example, a new team model that reduced burnout, a rapid mortality review that transformed team culture, and behavioral insights that shifted provider behavior. By making QI work relatable and emotionally resonant, the symposium created psychological engagement that traditional slide-based academic reporting often lacks, thereby enhancing both comprehension and application [9,34]. This approach is supported by research demonstrating that narrative formats enhance engagement, comprehension, and motivation to act by fostering sense-making and emotional resonance, key drivers of organizational learning and behavioral change [34]. 

Sessions linking behavioral economics, technology, and QI resonated strongly with participants. This reflected the health system's growing interest in applying behavioral science frameworks, nudge theory, and data analytics to strengthen evidence-based, equitable, and patient-centered care. The enthusiasm for these topics suggests increasing readiness among frontline clinicians and staff to adopt modern improvement tools and highlights the value of integrating behavioral science and digital health metrics into QI education.

Taken together, the symposium demonstrated that when content, format, and leadership engagement are intentionally aligned, health system-wide QI events can meaningfully influence knowledge, culture, and motivation. These insights can inform future efforts to scale learning, foster system-wide coherence in QI strategy, and strengthen the infrastructure needed for a continuously learning health system.

Limitations

There are several limitations that warrant consideration. First, this evaluation was conducted at a single academic health system and relied primarily on self-reported data, which may be subject to response and social desirability bias. Post-event surveys were optional and anonymous, increasing the possibility that respondents with higher satisfaction or engagement were more likely to complete evaluations. Second, the self-selection of attendees may have resulted in overrepresentation of individuals already engaged in or enthusiastic about quality improvement, limiting the generalizability of perceived learning outcomes. In addition, nurse attendees were invited to complete both a general evaluation and a nursing-specific survey required for CNE credit, whereas other participants completed only the general survey. Although it is possible that a small number of nurses completed the general survey, any resulting misclassification is likely minimal given the larger sampling frame of general attendees.

Finally, as a pragmatic program evaluation, this learning report did not include matched pre-post analyses, objective behavioral measures, or longitudinal or system-level outcomes. As such, findings should be interpreted as reflecting perceived learning, engagement, and readiness rather than demonstrated changes in clinical practice, quality outcomes, or organizational performance. Future iterations may build on this formative work by incorporating larger and more diverse samples, multi-site designs, and objective or longitudinal outcome measures (e.g., initiation or completion of QI projects, process or clinical metrics) to assess whether narrative-based educational symposia contribute to sustained changes in quality improvement capability and learning health system maturity.

## Conclusions

The inaugural DoM Quality Symposium demonstrated the power of convening an interprofessional community around a shared mission of continuous improvement. By combining leadership visibility, data-driven storytelling, and behavioral insight, the symposium supported learning and connection across UCLA Health and the broader healthcare community. Quantitatively, the event demonstrated high self-reported applicability and perceived learning among respondents, reinforcing the idea that intentionally designed, narrative-driven interprofessional learning events can support the development of QI capability across a complex academic health system.

National quality frameworks increasingly emphasize collective ownership of quality as foundational to healthcare excellence. The symposium provided a practical example of how intentional design, shared learning, and cross-disciplinary engagement can operationalize this principle in practice. As a health system-level educational intervention, this model illustrates how local learning initiatives may align institutional priorities with national quality goals. While the observed outcomes reflect perceived learning rather than demonstrated system-level change, such events may serve as important enabling platforms for cultural alignment and engagement. Future iterations will focus on strengthening sustainability, broadening participation across roles and sites, and incorporating longitudinal evaluation to better understand how educational symposia contribute to sustained improvement and learning health system maturity.

## Appendices

Appendix A. DoM Quality Symposium Pre-Event Questionnaire

We're excited to welcome you to the inaugural Department of Medicine (DoM) Quality Symposium: "The Pulse of Progress: Why Quality Is Healthcare's Vital Sign." The symposium will bring together thought leaders, clinicians, staff, and trainees to explore why quality is healthcare's most critical vital sign. Through TED-style presentations, keynote insights, and interactive discussions, participants will discover how innovative strategies, behavioral science, and data-driven approaches are shaping the future of care delivery. The program highlights real-world examples from ambulatory and inpatient settings and showcases advances in care delivery, research, and education. Attendees will leave with actionable insights to strengthen systems and foster collaboration across UCLA Health and beyond.     

📍 Ronald Reagan UCLA Medical Center, Tamkin Auditorium 📅 Wednesday, October 22, 2025

 🕗 8:00 AM - 1:00 PM     

➡️ Registration is free but required. Please RSVP to confirm your attendance and help us plan a meaningful and engaging experience for all participants.     For details on speakers, sessions, and topics, please visit the symposium agenda at www.uclahealth.org/qualitysymposium. 

Q1 First Name

________________________________________________________________

Q2 Last Name

________________________________________________________________

Q3 Email address

________________________________________________________________

Q4 Please indicate your academic degree(s) or professional designation(s) (e.g., PhD, MD, RN, MBA, CPA), if applicable:

________________________________________________________________

Q5 Are you a UCLA employee?

o Yes  

o No   

Q6 What organization are you affiliated with?

________________________________________________________________

Q7 Please indicate your role at UCLA

o Faculty

o Staff

o Trainee

o Other __________________________________________________

Q8 Are you interested in receiving Continuing Nursing Education (CNE) credit for attending this symposium?

o Yes

o No

o Unsure

Q9 How did you hear about this opportunity?

o Email

o Organization

o Peer

o Other (please specify)

__________________________________________________

Q10 Is there an organization or individual you recommend we send this invitation to?

________________________________________________________________

Q11 Have you ever been involved in a quality improvement (QI) project?

o Yes, I’ve led a QI project 

o Yes, I’ve participated in a QI project 

o Not yet, but I’m interested 

o No, and I’m not sure

Q12 How familiar are you with QI methodology and tools (e.g., PDSA cycles, root cause analysis, fish bone diagrams, etc.)?

o Very familiar - I use them regularly

o Somewhat familiar - I've been exposed but don't use them often

o Not familiar - this is new to me

Q13 What motivates you to attend this symposium?

▢        Learn new tools/strategies 

▢        Hear about successful projects

▢        Network with colleagues

▢        Explore opportunities to get involved in QI

▢        Learn about current UCLA Health QI initiatives

▢        Other (please specify)

__________________________________________________

Q14 Do you have any questions or comments you'd like to share?

________________________________________________________________

________________________________________________________________

________________________________________________________________

________________________________________________________________

________________________________________________________________

Appendix B. DoM Quality Symposium - Post-Event Questionnaire

Thank you for attending the inaugural Department of Medicine (DoM) Quality Symposium, “The Pulse of Progress: Why Quality Is Healthcare’s Vital Sign.” We value your feedback and invite you to share your thoughts about your experience. Your responses will help us enhance future programs and continue building a culture of quality improvement across UCLA Health and beyond. The goal of the symposium was for participants to identify and apply 1-3 actionable insights aligned with the following learning objectives:               

·       Recognize why quality is a core driver of healthcare outcomes and organizational performance in today’s evolving landscape. 

·       Explore innovative strategies and behavioral best practices successfully implemented across care settings.       

·       Engage with peers to exchange ideas, foster collaboration, and build capacity for advancing quality and continuous improvement in healthcare delivery.

Q1 How would you rate your overall experience at the DoM Quality Symposium?

o Excellent  

o Good  

o Average  

o Poor

o Terrible

Q2 Which parts of the symposium did you find most valuable?

▢        Keynote address (Dr. Nasim Afsar) 

▢        Ambulatory Care Quality presentations 

▢        Inpatient Care Quality presentations 

▢        Research & Education presentations 

▢        Lunch & Networking opportunities 

▢        Other

Q3 If  “Other,” please specify

________________________________________________________________

Q4 The symposium increased my understanding of how quality improvement impacts patient outcomes and organizational performance.

o Strongly agree  

o Agree  

o Neutral

o Disagree   

o Strongly disagree  

Q5  I gained at least one actionable insight or idea that I can apply in my own work.

o Yes  

o Somewhat  

o No   

Q6 If you selected “Yes” or “Somewhat,” please describe the insight or idea you found most valuable and how you plan to apply it in your work. 

________________________________________________________________

Q7 Please share any feedback on how we can make the DoM Quality Symposium even more impactful in the future.

________________________________________________________________

Q8 If you are interested in being entered into a drawing to receive a copy of Dr. Nasim Afsar’s new book, please provide your email.

________________________________________________________________
